# Psychiatric Antecedents in Adolescents at Clinical High Risk for Psychosis: Insights from the “Parma At-Risk Mental States” Follow-up Program

**DOI:** 10.1007/s10802-025-01368-0

**Published:** 2025-08-30

**Authors:** Lorenzo Pelizza, Fabio Catalano, Emanuela Leuci, Emanuela Quattrone, Derna Palmisano, Simona Pupo, Giuseppina Paulillo, Clara Pellegrini, Pietro Pellegrini, Marco Menchetti

**Affiliations:** 1 Department of Biomedical and Neuromotor Sciences, Alma Mater Studiorum – Università di Bologna, Bologna (BO), Italy; 2 Department of Mental Health and Pathological Addiction, Azienda USL di Parma, Parma (PR), Italy; 3 Pain Therapy Service, Department of Medicine and Surgery, Azienda Ospedaliero-Universitaria di Parma, Parma (PR), Italy; 4 Lorenzo Pelizza c/o Istituto di Psichiatria “Paolo Ottonello” - viale Pepoli, 5 - 40126 Bologna (BO), Italy

**Keywords:** Clinical high risk, Antecedents, Early psychosis, Early intervention in psychosis, Outcome, Help-seeking

## Abstract

Exploring psychiatric antecedents in youths at Clinical High Risk for Psychosis (CHR-P) could help understand determinants for early detection and timely care pathways, consequently improving outcomes and prognosis. The aims of this investigation were: (1) to examine the proportion of CHR-P individuals with past contact with mental healthcare services enrolled within a specialized CHR-P service, and (2) to longitudinally compare sociodemographic, clinical, and treatment parameters between CHR-P subjects with and without psychiatric antecedents across 2 years of follow-up. All participants (aged 12–25 years) were recruited within the “Parma At Risk Mental States” (PARMS) program. Across the follow-up, they completed the Health of the Nation Outcome Scale (HoNOS) and the Positive And Negative Syndrome Scale (PANSS). A mixed-design ANOVA and a Kaplan-Meier survival analysis were also used. Of the 170 CHR-P subjects, 95 (55.9%) had previous contact with mental health services. In 85.2% of cases, a care discontinuity was observed. Main psychiatric antecedents were learning disorders (7.4%), anxiety disorder (23.1%), conduct disorder (22.1%), and depressive disorder (14.7%). CHR-P youths with psychiatric antecedents had longer duration of untreated psychosis and a higher 2-year incidence rate of psychosis transition. Having previous contact with psychiatric services also resulted to be a associated with poorer social functioning over time. Carefully monitor mental health suffering and related help-seeking-behavior in youths typically manifested in their early 20’s is crucial, also in terms of psychosis prevention. Moreover, special attention should be given to service engagement as care continuity within adolescent-adult transition.

**Supplementary Information:**

The online version contains supplementary material available at 10.1007/s10802-025-01368-0.

## Introduction

While there’s sufficient evidence on psychiatric antecedents in psychosis and schizophrenia (e.g., anxiety, depressive symptoms, attenuated psychotic symptoms, disruptive behaviors, sleep problems, basic symptoms/schizotypy) (Rutter et al., 2006; Uher et al., 2024), very little is known about antecedents in adolescents at “Clinical High Risk for Psychosis” (CHR-P) (Pelizza et al., 2025a). Since psychoses (and schizophrenia, in particular) are now increasingly considered as neurodevelopmental disorders, identifying early subclinical/clinical symptoms and syndrome of CHR-P mental states is crucial, as they could suggest psychopathological continuity and may interact with other risk factors synergistically increasing the risk of later developing psychosis (Cannon et al., 2002; Welham et al., 2009). From this point of view, CHR-P period could be considered a relatively late phase of an earlier developmental derailment (Rapoport et al., 2012; Poletti et al., 2019). Unfortunately, a recent meta-analysis of prospective investigations indicated that the association of psychiatric antecedents with transition in CHR-P studies was numerically weaker (Solmi et al., 2023a). In particular, for the two more commonly reported psychopathological antecedents (i.e., anxiety and depressive symptoms), a meta-regression showed a reduced effect size of the predictor-outcome association, controlling for antecedent type (Uher et al., 2024).

Overall, there is a lack of knowledge about childhood-onset disorders in prodromal teens, even though many risk factors for psychosis and schizophrenia occur during infancy (Mazzoni et al., 2009). Specifically, to date, most clinical antecedents (such as depression and anxiety) appear to be trans-diagnostic (Solmi et al., 2023b) and presented relevant associations with the onsets of major depressive, bipolar, and psychotic disorders. Furthermore, given the low number of prospective studies, the CHR-P design appears to underestimate the strength of antecedent-onset relationships. However, biological and familial investigations reported interesting findings. In this respect, a neurobiological review showed specific structural abnormalities, especially in prefrontal cortex and hippocampus, in 7 years of age children with genetic burden for schizophrenia (Nanda et al., 2014). Moreover, polygenic risk scores seem to account for about 80% of the variation in the risk of psychosis (Sullivan et al., 2003). In offspring of individuals with schizophrenia, developmental abnormalities during childhood frequently precede the onset of symptoms, especially attentional and behavioral impairments, social difficulties, and motor abnormalities (Erlenmeyer-Kimling, 2001).

Furthermore, the importance of further elucidating on the premorbid phase in CHR-P mental states is also accentuated by recent empirical investigations suggesting that poor premorbid academic functioning, poor social adjustment, and the presence of neurodevelopmental disorders (e.g., childhood repetitive behavior and attention-deficit/hyperactivity disorder [ADHD]) may act as predictors of conversion to psychosis (Tarbox et al., 2013; Dannevang et al., 2018; Jutla et al., 2020). Specifically, in an interesting cohort study by Welham and colleagues (2009), maternal reports related to general psychopathology, social and attentional dysfunction, aggression or delinquency at years 5 and 14 showed significant association with non-affective psychosis in youths by year 21. Child trauma is also a crucial risk factor for psychosis worth to be investigated as antecedent in CHR-P subjects, showing a prevalence rate around 60% and a risk higher of 2.72 times than healthy individuals (Varese et al., 2012; Loewy et al., 2019).

Additionally, the “Jerusalem Infant Development Study” found a 25% prevalence rate of affective and anxiety disorders and a 37% prevalence rate of disruptive behavior disorders in the group at high genetic risk for psychosis (Hans et al., 2000). Other research examining diagnoses during prodromal phase of psychosis showed prevalence rates around 50% for mood disorders, 30% for anxiety disorders, 35% for substance use disorders (Meyer et al., 2005; Rosen et al., 2006), but with small attention to childhood onset disorders. Finally, in a case series of 9 teens (aged 13–17 years) identified as prodromal to psychosis (Mazzoni et al., 2009), childhood-onset diagnoses commonly endorsed ADHD (5/9), oppositional defiant disorder (5/9), and separation anxiety (3/9).

In summary, to our knowledge, very few studies systematically investigated psychiatric antecedents and past specialist contact in young people at CHR-P, especially from a prognostic point of view and as predictors of treatment response and long-term outcomes. In this neurodevelopmental framework, we believe that better knowing and correctly identifying the most common psychiatric antecedents of CHR-P mental states is very important to avoid their psychopathological underestimation by clinicians, to counteract care discontinuity over time, and to longitudinally monitor symptoms and syndromes that, although trans-diagnostic, may be predictive of severe mental illness (such as psychotic, bipolar, and major depressive disorders). Together with the “pluripotent” CHR-P features (Lo Buglio et al., 2024), this would allow us to illustrate an “early psychopathology” that requires great attention in the context of prevention/early intervention of serious mental disorders. Therefore, the specific aims of this examination were: (a) to investigate psychiatric antecedents in a young CHR-P population treated within a specialized CHR-P service, and (b) to determine whether these antecedents could be considered a predictor of poor clinical or functional outcome during a 2 years follow-up period.

## Methods

### Participants and Setting

All participants were enrolled within the “Parma At-Risk Mental States” (PARMS) program between January 2016 and December 2022. The PARMS is a specialized “Early Intervention in Psychosis” (EIP) service for young people at CHR-P that was implemented in all community adolescent and adult mental healthcare centers in the Parma Department of Mental Health (Northern Italy) (Pelizza et al., 2023a).

For the specific goals of this examination, inclusion criteria were: (a) specialist help-seeking request, (b) enrollment in the PARMS program, (c) age 12–25 years, (d) to meet CHR-P criteria at entry as defined in the “Comprehensive Assessment of At-Risk Mental States” (CAARMS): i.e., “Brief Limited Intermittent Psychotic Symptoms” (BLIPS), “Genetic Vulnerability” (GV), or “Attenuated Psychotic Symptoms” (APS) (Yung et al., 2005). As exclusion criteria, we considered: (1) previous exposure to antipsychotic (AP) drug or current AP exposure for more than 4 weeks, (2) history of past overt psychotic episode, (3) other neurological or medical condition manifesting with psychiatric symptoms, and (4) known intellectual disability (i.e., IQ < 70). Previous AP exposure (i.e., in past episode before the PARMS recruitment) was here treated as a proxy for previous psychotic phase according to the original CAARMS psychosis threshold criteria (i.e., “…essentially that at which AP medication would probably be started in the common clinical practice”) (Pelizza et al., 2019). A current AP exposure for more than 4 weeks was also considered to minimize pharmacological interference with our psychopathological assessment at presentation (Poletti et al., 2022a; Di Lisi et al., 2024).

### Assessment and Measures

The presence of CHR-P at presentation was detected using the psychometric criteria of the “Comprehensive Assessment of At-Risk Mental States” (CAARMS), approved Italian version (Raballo et al., 2013). The clinical interview was administered by specifically trained PARMS team members. Scoring workshops and supervision sessions were also regularly conducted to ensure excellent inter-rater reliability values (Intra-Class-Correlation [ICC] coefficients > 0.924) (Paterlini et al., 2019).

The clinical assessment also included the Social and Occupational Functioning Assessment Scale (SOFAS) scale (Yung et al., 2005), the Positive And Negative Syndrome Scale (PANSS) (Kay et al., 1987), and the Health of the Nation Outcome Scale (HoNOS) (Wing et al., 1999).

The SOFAS is a widely used instrument for the assessment of daily social and occupational functioning in individuals with early psychosis, including youths at CHR-P (McHugh et al., 2018). In this research, we used the Italian adaptation of the SOFAS included in the CAARMS, which showed good psychometric properties (ICC coefficient = 0.974) (Poletti et al., 2021). As index of functional remission, we used a SOFAS score of ≥ 61 (Spellmann et al., 2012).

The HoNOS is a scale specifically developed to assess social and clinical outcomes in people with severe mental illness, including early psychosis (Pelizza et al., 2024a). It was specifically validated as routine measure of outcome in adolescents and young adults enrolled in EIP programs (Preti et al., 2012; Penno et al., 2017). As indicated by Wing and co-workers (1998), we considered four main outcome domains: psychiatric symptoms, impairment, social problems, and behavioral problems. In this investigation, we administered the Italian version of the HoNOS, which showed good to excellent reliability and validity values (r coefficients > 0.420 [*p* < 0.001]) (Gigantesco et al., 2007). As further index of functional remission, we also used scores of ≤ 2 simultaneously in HoNOS items 9, 10, and 11 (Kortrijk et al., 2012; Pelizza et al., 2025b).

The PANSS is a widely used scale that assesses psychopathology in individuals with psychosis, including its early phase (Yang et al., 2018; Pelizza et al., 2023b). As proposed by Shafer and Dazzi (2019) in their meta-analysis on the PANSS factor structure, we included the following five main dimensions: “Disorganization”, “Positive Symptoms”, “Negative Symptoms”, “Affect” (anxious-depressive), and “Resistance/Excitement-Activity”. In this examination, we used the Italian version of the PANSS which had good psychometric properties (r coefficients > 0.457 [*p* < 0.001]) (Pancheri et al., 1995). As index of symptomatic remission, we used scores of ≤ 3 simultaneously in PANSS item P1, P2, P3, N1, N4, N6, G5, G9 (Andreasen et al., 2005).

Finally, an anamnestic chart was also completed at entry and along the follow-up period to capture a broad range of sociodemographic, clinical, and outcome parameters including gender, age at presentation, years of education, civil and living status, occupation, ethnicity and migration, source of referral, past specialist contact and psychiatric antecedents, “Duration of Untreated Prodromal Symptoms” (DUPrS), family history of psychosis, hospitalizations, current substance abuse, service disengagement, suicide attempt, psychosis transition, current suicidal ideation, functional recovery, CHR-P criteria persistence, psychopharmacological treatment, and PARMS specialized psychosocial interventions. Specifically, the DUPrS was defined as the time interval between the onset of the first attenuated psychotic positive symptom (at a minimum moderate level, corresponding to a score of 3 or higher on the CAARMS) and the commencement of professional assistance at mental healthcare services (Zhang et al., 2023). Current suicidal ideation was intended as “expressed desire, intent or actions to harm or kill self”, corresponding to an item 4 score of ≥ 2 in the Brief Psychiatric Rating Scale (BPRS): i.e., at least “occasional suicidal thoughts without intent or specific plan or he/she feels they would be better off dead (Shafer et al., 2017). Functional recovery was simply defined as return at school/work (Silva & Restrepo, 2019).

### Procedures

After CAARMS interviews, all participants were divided into CHR-P + or CHR-P- subgroups depending on having or not a past specialist contact for mental problems (psychiatric antecedents) (Biancalani et al., 2024). They were then tested for clinical and outcome parameters with PANSS, HoNOS and SOFAS scales both at presentation and every 12 months along the 2 years of follow-up by trained PARMS team members. Moreover, 2-year incidence rates of service disengagement, psychosis transition, new hospitalization, and new suicide attempt were also calculated and compared between the two CHR-P subgroups. Inter-group comparisons in terms of functioning, psychopathological, and outcome scores during the follow-up period were also examined, as well as inter-group differences in incidence rates of AP prescription, suicidal ideation, CHR-P criteria persistence, functional recovery, and symptomatic remission.

As for specialized EIP treatments, the PARMS protocol provided a 2-year comprehensive package including multi-component psychosocial interventions (combining individual psychotherapy based on cognitive-behavioral approach, psychoeducational sessions for family members, and a recovery-oriented case management), together with pharmacotherapy (where appropriate), according to the current EIP guidelines (Schmidt et al., 2015; Addington et al., 2017). Specifically, AP medication was avoided unless frank positive psychotic symptoms were sustained for at least 1 week, or briefer/milder positive symptoms were directly associated with a high risk of self-harm/aggression, or when sub-threshold positive symptoms persisted despite psychotherapy and other psychosocial intervention and were causing distress and/or increasing functional disability (Poletti et al., 2024). In these cases, psychosocial treatments could be complemented by low-dose second-generation antipsychotics (Pelizza et al., 2025c).

All subjects and their parents (if minors) agreed to participate to the research and gave their written informed consent prior to their inclusion in the study. Local ethics approval has been obtained for the investigation (AVEN Ethics Committee: protocol n. 559/2020/OSS*/AUSLPR). This examination was carried forward in agreement with the Code of Ethics of the World Medical Association (1964 Declaration of Helsinki and its later amendments).

### Statistical Analysis

Collected data were analyzed using the Statistical Package for Social Science (SPSS) 28.0 for Windows (IBM Corp., 2021). All tests were two-tailed, with a significance level set at 0.05. The Bonferroni p correction was applied to control multiple comparisons and for the probability of committing type I errors (Curtin & Schulz, 1998). There were no missing data. In intergroup-group comparisons, the Chi-square (Χ^2^) test for categorical variables and the Mann-Whitney U test for continuous variables were used (Neely et al., 2003). As for outcome parameters that had as endpoint the time when a specific event occurred, we performed the Kaplan-Meier survival analysis, especially to consider the different duration of follow-ups and participants who dropped out from the study protocol (Gomes et al., 2024). As for outcome parameters that did not have as endpoint the time when a specific event occurred, we performed a binary logistic regression analysis calculating hazard risk (Bender & Grouven, 1997). Finally, a mixed-design ANOVA analysis was performed to evaluate the temporal stability of HoNOS, PANSS, and SOFAS scores within and between the two CHR-P subgroups across the 2-year follow-up period (Blanca et al., 2023).

## Results

**Fig. 1:**
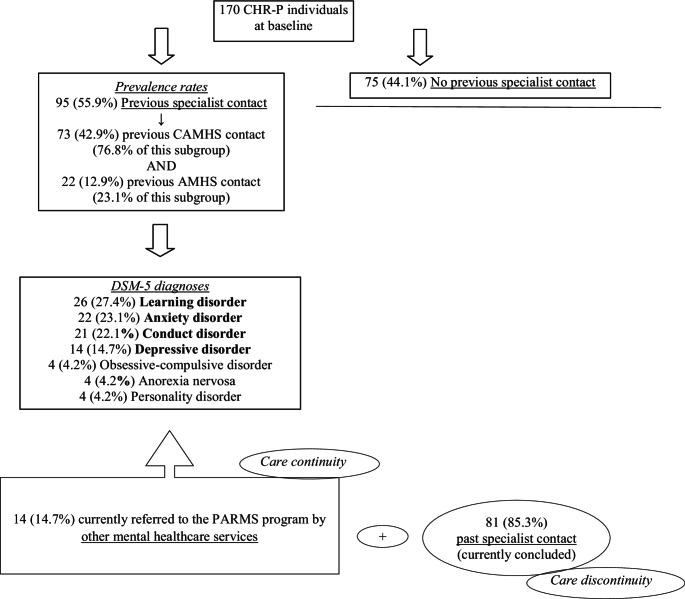
Prevalence rate of CHR-P individuals with and without previous specialist contact. CHR-P = Clinical High Risk for Psychosis, CAMHS = Child and Adolescent Mental Health Services, AMHS = Adult Mental Health Services; DSM-5 = Diagnostic and Statistical Manual of mental disorders, 5th edition; PARMS = Parma At-Risk Mental States

A total of 170 CHR-P individuals were recruited in this research through the PARMS program. Of them, 95 (55.9%) had a previous specialist (mental healthcare) contact and were included in the CHR-P + subgroup. The remaining 75 subjects were grouped in the CHR-P- subsample (Fig. 1).

In the CHR-P + group, 73 participants (76.8%) had a past contact within child/Adolescent Mental Health Services (CAMHSs), while only 22 (23.1%) within Adult Mental Health Services (AMHSs). Their age at presentation was 19.62 ± 6.42 years compared to an age at previous contact of 16.30 ± 4.57 years. The main DSM-5 diagnoses at the earlier contact were learning disorder (*n* = 26, 27.4%), anxiety disorder (*n* = 22, 23.1%), conduct disorder (*n* = 21, 22.1%), and depressive disorder (*n* = 14, 14.7%). The conduct disorder diagnostic entity also encompassed hyperkinetic disorders. Finally, only 14 individuals (14.7% of the CHR-P + subsample) were directly referred to the PARMS program by other mental health services within a clinical pathway of care continuity. In the remaining 81 (85.2%) subjects, their past specialist contact ended with their care retention in mental health services being terminated (care discontinuity).

Baseline clinical and sociodemographic comparisons between the two CHR-P subgroups are shown in the Table 1. Compared to CHR-P-, CHR-P participants with previous specialist contact had a longer DUPrS. No other inter-group differences in terms of socio-demography, psychopathology, functioning, treatment and clinical characteristics were found.

**Table 1:** Baseline sociodemographic and clinical comparisons in the two CHR-P subgroups (n = 170)

Variable	CHR-P + (n = 95)	CHR-P- (n = 75)	X^2^/z	p
Gender (females)	48 (49.5%)	41 (54.7%)	.453	.501
Citizenship (Italian)	84 (88.4%)	61 (81.3%)	1.679	.195
*Ethnic group*			3.896	.420
White	87 (91.6%)	62 (82.7%)	[1.8]	
Black	2 (2.1%)	2 (2.7%)	[-.2]	
Asian	1 (1.1%)	3 (4.0%)	[-1.3]	
North African	3 (3.2%)	6 (8.0%)	[-1.4]	
Hispanic	2 (2.1%)	2 (2.7%)	[-.2]	
Migrant Status	11 (11.6%)	14 (18.7%)	1.679	.195
Age (at entry)	19.62 ± 6.42	19.40 ± 5.54	.381	.703
Educational level (in years)	11.29 ± 2.20	11.47 ± 2.54	.108	.914
*Civil status*			.293	.695
Single	91 (95.8%)	73 (97.3%)	[-.5]	
Married/cohabitant	4 (4.2%)	2 (2.7%)	[.5]	
*Living status*			1.328	.723
Alone	3 (3.2%)	3 (4.0%)	[-.3]	
Living with partner	6 (6.3%)	3 (4.0%)	[.7]	
Living with parents	85 (89.5%)	69 (92.0%)	[-.6]	
Other cohabitation	1 (1.1%)	0 (0.0%)	[.9]	
*Occupation*			4.152	.125
NEET	31 (32.6%)	16 (21.3%)	[1.6]	
Student	57 (60.0%)	48 (64.0%)	[-.5]	
Employed	7 (7.4%)	11 (14.7%)	[-1.5]	
*Source of referral*			1.864	.868
Primary care	34 (35.8%)	24 (32.0%)	[.5]	
Family members	12 (12.6%)	8 (10.7%)	[.4]	
Self-referral Emergency room	13 (13.7%) 11 (11.6%)	11 (14.7%) 14 (18.7%)	[-.2] [-1.3]	
School/Social services	11 (11.6%)	8 (10.7%)	[.2]	
Other mental healthcare services	14 (14.7%)	10 (13.3%)	[.3]	
DUPrS (in weeks)	73.54 ± 55.20	58.62 ± 53.13	-1.869	**.042**
Family history for psychosis	38 (40.0%)	22 (29.3%)		.385
Baseline hospitalization	13 (13.7%)	9 (12.0%)	.755	.745
Previous suicide attempts	8 (8.4%)	7 (9.3%)	.106	.835
Substance abuse	14 (14.7%)	12 (16.0%)	.043	.820
*Treatments*			.052	
Baseline AP prescription	39 (41.1%)	34 (45.3%)		.576
Baseline AD prescription	29 (30.5%)	21 (28.0%)	.313	.720
Baseline MS prescription	10 (10.5%)	6 (8.0%)	.129	.575
Baseline BDZ prescription	26 (27.4%)	18 (24.0%)	.314	.619
Baseline acceptance of individual psychotherapy proposal	47 (56.0%)	44 (63.4%)	.248	.327
Baseline acceptance of family psychoeducation proposal	44 (50.6%)	33 (47.8%)	.960	.733
Baseline acceptance of case management proposal	45 (51.7%)	30 (43.5%)	.116	.306
*Baseline CHR-P subgroup*			1.048	
APS	73 (76.8%)	58 (77.3%)		.940
GV	8 (8.4%)	6 (8.0%)	.006	.921
BLIPS	14 (14.7%)	11 (14.7%)	.010	.990
*Baseline HoNOS scores*			.000	
Behavioral problems	2.11 ± 1.87	2.61 ± 1.92		.086
Impairment	1.73 ± 1.43	1.57 ± 1.49	-1.718	.377
Psychiatric symptoms	8.46 ± 3.37	8.08 ± 2.96		.564
Social problems	5.34 ± 2.98	4.57 ± 3.06	-.884	.082
HoNOS total score *Baseline PANSS scores*	17.63 ± 5.63	16.84 ± 5.88	-.576 -1.737 -.583	.560
Positive symptoms	10.79 ± 4.15	10.13 ± 3.64		.384
Negative symptoms Disorganization	17.05 ± 7.06 15.51 ± 5.94	18.17 ± 7.67 14.88 ± 5.31	-.870 -1.083	.279 .592
			-.537	
Affect	14.65 ± 5.06	14.84 ± 5.40	-.228	.819
Resistance/Excitement-activity	7.24 ± 3.33	7.31 ± 3.38	-.111	.911
	67.34 ± 19.30	67.63 ± 17.03	-.487	.629
PANSS total score				
G12 “Lack of judgment/insight”	2.09 ± 1.32	2.29 ± 1.51	-.731	.465
SOFAS score	49.43 ± 7.53	48.79 ± 8.56	-.341	.733

CHR-P = Clinical High Risk for Psychosis; CHR-P + = CHR-P individuals with previous specialist contact; CHR-P- = CHR-P individuals without previous specialist contact; NEET = Not [engaged] in Education, Employment or Training; DUPrS = Duration of Untreated Prodromal Symptoms; AP = Antipsychotic medication; AD = Antidepressant medication; MS = Mood Stabilizer; BDZ = Benzodiazepine; APS = Attenuated Psychotic Symptoms; GV = Genetic Vulnerability; BLIPS = Brief Limited Intermittent Psychotic Symptoms; HoNOS = Health of the Nation Outcome Scale; PANSS = Positive And Negative Syndrome Scale; SOFAS = Social and Occupational Functioning Assessment Scale. Frequencies (and percentages) and mean ± standard deviation are reported. Chi-square (X^2^) test (or Fisher exact test, where appropriate) and Mann–Whitney U (z) test values are reported. Standardized adjusted residuals are in square brackets. Statistically significant p values are in bold. Bonferroni corrected p values are reported

**Fig. 2:**
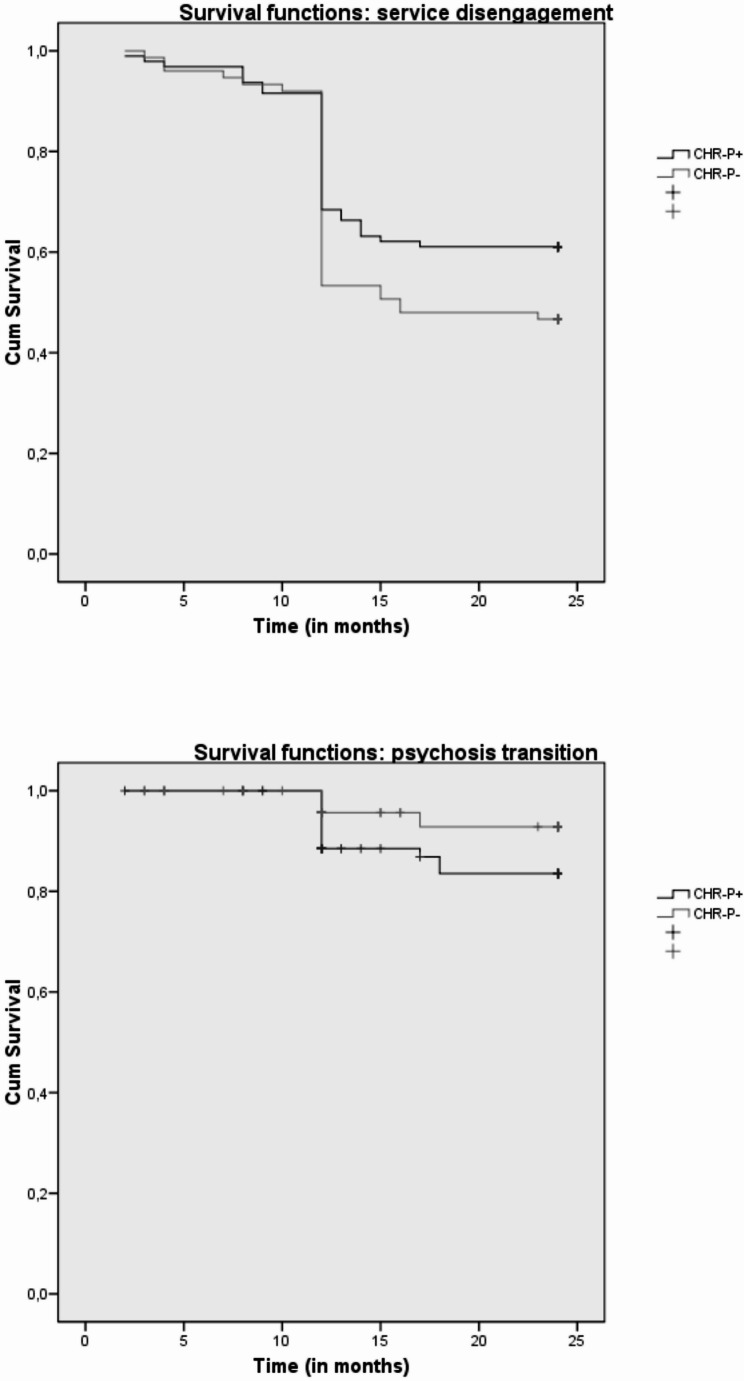
Kaplan-Meyer survival functions: comparisons on statistically significant 2-year time-to-event outcome incidence rates between the two CHR-P subgroups. CHR-P = Clinical High Risk for Psychosis; CHR-P + = CHR-P individuals with previous specialist contact; CHR-P- = CHR-P individuals without previous specialist contact

Notably, our Kaplan–Meier survival analysis results showed that CHR-P + participants had a lower 2-year incidence rate of service disengagement (38.9% vs. 53.3%) and a higher 2-year incidence rate of psychosis transition (16.5% vs. 7.2%) in comparison with CHR-P- individuals (Table 2; see also Fig. 2 for details on survival functions). No between-group differences in incidence rates of new hospitalization and new suicide attempt were observed across the follow-up period.

**Table 2:** Kaplan–Meier survival analysis results: comparisons on 2-year time-to-event outcome incidence rates among the two CHR-P subgroups

CHR-P subgroup	Number of events	1-cumulative proportion surviving at the time		Mean (in months) for 2-year *service disengagement* incidence rate				
		Estimate	SE	Estimate	SE	95% CI		
						Lower bound	Upper bound	
CHR-P + CHR-P- (Overall)	37 31 68	.389 .533 -	.050 .058 -	19.021 17.453 18.329	.668 .758 .505	17.712 15.967 17.340	20.330 18.940 19.319	
				Χ^2^	df	p		
Log Rank (Mantel-Cox)				3.161	1	**.045**		
CHR-P subgroup	Number of events	1-cumulative proportion surviving at the time		Mean (in months) for 2-year *new hospitalization* rate				
		Estimate	SE	Estimate	SE	95% CI		
						Lower bound	Upper bound	
CHR-P + CHR-P- (Overall)	8 7 15	.092 .129 -	.031 .048 -	22.897 22.765 22.857	.372 .441 .281	22.168 21.901 22.308	23.625 23.629 23.407	
				Χ^2^	df	p		
Log Rank (Mantel-Cox)				.063	1	.801		
CHR-P subgroup	Number of events	1-cumulative proportion surviving at the time		Mean (in months) for 2-year *new suicide attempt* incidence rate				
		Estimate	SE	Estimate	SE	95% CI		
						Lower bound		Upper bound
CHR-P + CHR-P- (Overall)	4 2 6	.052 .043 -	.026 .031 -	23.479 23.629 23.551	.256 .258 .180	22.978 23.123 23.198		23.980 24.135 23.903
				Χ^2^	df	p		
Log Rank (Mantel-Cox)				.206	1	.650		
CHR-P subgroup	Number of events	1-cumulative proportion surviving at the time		Mean (in months) for 2-year *psychosis transition* incidence rate				
		Estimate	SE	Estimate	SE	95% CI		
						Lower bound	Upper bound	
CHR-P + CHR-P- (Overall)	13 4 17	.165 .072 -	.043 .037 -	21.305 23.281 22.728	.486 .349 .291	20.451 22.598 21.157	22.160 23.964 22.799	
Log Rank (Mantel-Cox)				Χ^2^	df	p		
				3.123	1	**.048**		

CHR-P = Clinical High Risk for Psychosis; CHR-P + = CHR-P individuals with previous specialist contact; CHR-P- = CHR-P individuals without previous specialist contact; SE = Standard Error; 95% CI = 95% Confidence Intervals for estimate; Log Rank = Logarithm Rank Test; X^2^ = Chi-Square test; df = degrees of freedom; p = statistical value. Statistically significant p values are in bold

Our binary logistic regression analysis results for 2-year not time-to-event outcome parameters in the two CHR-P subgroups showed no statistically relevant inter-group differences in terms of hazard ratio values for suicidal ideation, functional recovery, SOFAS and functional remission, PANSS symptomatic remission, CHR-P criteria persistence, and AP prescription rate along the follow-up period (Table 3). Conversely, our mixed-design ANOVA analysis results showed a statistically significant effect of time on all HoNOS, PANSS, and SOFAS scores, as well as on equivalent AP doses (Table 4). Specifically, we observed longitudinal improvements in outcome, psychopathological and functioning severity levels, as well as a reduction in AP dosage over time. However, statistically significant group effects for HoNOS “Social problems” subscores and HoNOS total scores were notably found. Specifically, compared to CHR-P-, CHR-P + individuals maintained higher scores in these HoNOS dimensions along the 2 years of follow-up (see also Supplementary Materials [Figure S1] for details on mixed-design ANOVA profile plots).

**Table 3:** Binary logistic regression analysis results for 2-year not time-to-event outcome variables in the two CHR-P subgroups

Dependent variable (n = 156)	CHR-P + (n = 87)	CHR-P- (n = 69)	Statistic test				
			B (SE)	HR	95% CI		p
					Lower Higher		
1-year current suicidal ideation 1-year functional recovery	19 (21.8%) 60 (69.0%)	18 (26.1%) 48 (69.6%)	-.234 (.377) .028 (.350)	.792 1.029	.378 .518	1.659 2.040	.536 .936
1-year SOFAS functional remission	52 (59.8%)	49 (71.0%)	.500 (.344)	1.649	.841	3.235	.146
1-year HoNOS functional remission	65 (74.7%)	54 (78.3%)	.198 (.382)	1.218	.576	2.577	.605
1-year PANSS symptomatic remission	58 (66.7%)	48 (69.6%)	.134 (.347)	1.143	.579	2.225	.700
1-year CHR-P criteria persistence	30 (34.5%)	19 (27.5%)	-.326 (.351)	.722	.363	1.438	.576
1-year AP prescription rate	37 (42.5%)	21 (30.4%)	-.505 (.340)	.604	.310	1.177	.604
Dependent variable (n = 102)	CHR-P + (n = 58)	CHR-P- (n = 44)	Statistic test				
			B (SE)	HR	95% CI		p
					Lower Higher		
2-year current suicidal ideation	9 (15.5%)	9 (20.4%)	-.395 (.522)	.673	.242	1.875	.449
2-year functional recovery	44 (75.9%)	30 (68.1%)	-.343 (.453)	.710	.292	1.726	.450
2-year SOFAS functional remission	38 (65.6%)	33 (75.0%)		1.579	.661	3.773	.304
2-year HoNOS functional remission	46 (79.3%)	37 (84.1%)	.457 (.444)	1.565	.535	4.575	.413
2-year PANSS symptomatic remission	37 (63.8%)	34 (77.2%)	.448 (.547)	1.816	.746	4.420	.172
2-year CHR-P criteria persistence	19 (38.2%)	8 (18.1%)	.597 (.454)	.483	.188	1.243	.131
2-year AP prescription rate	25 (43.1%)	16 (36.4%)	-.728 (.482) -.208 (.414)	.812	.361	1.828	.615

CHR-P = Clinical High Risk for Psychosis; CHR-P + = CHR-P individuals with previous specialist contact; CHR-P- = CHR-P individuals without previous specialist contact; SOFAS = Social and Occupational Functioning Assessment Scale; HoNOS = Health of the Nation Outcome Scale; PANSS = Positive And Negative Syndrome Scale; AP = Antipsychotic; B = regression coefficient, SE = Standard Error; HR = Hazard Ratio; 95% CI = 95% confidence intervals for HR; p = statistical significance. Significant statistical p values are in bold. Cumulative incidence rates are also reported. Current suicidal ideation = BPRS item 4 score of ≥ 2; Functional recovery = return to work/school; SOFAS functional remission = SOFAS score of ≥ 60; HoNOS functional remission = HoNOS item 9, 10, and 11scores of ≤ 2; PANSS symptomatic remission = PANSS item P1, P2, P3, N1, N4, N6, G5, G9 scores of ≤ 3

**Table 4:** Mixed-design ANOVA results: psychopathological and outcome parameters across the 2-year follow-up period in the two CHR-P subgroups

Variable	Time effect				Group effect				Interaction effect (time x group)			
	df	F	p	η^2^	df	F	p	η^2^	df	F	p	η^2^
*HoNOS scores*												
Behavioral problems	1.4	51.961	**.001**	.349	1	.689	.405	.005	1.4	.449	.571	.005
Impairment	2	18.272	**.001**	.157	1	1.601	.209	.016	2	2.034	.134	.020
Psychiatric symptoms	1.6	92.461	**.001**	.485	1	1.819	.181	.018	1.6	.050	.923	.001
Social problems	1.8	49.256	**.001**	.334	1	8.543	**.004**	.080	1.8	.139	.849	.001
HoNOS total score	1.6	98.526	**.001**	.504	1	4.178	**.044**	.041	1.6	.245	.738	.003
*PANSS scores*												
Positive symptoms	1.5	19.261	**.001**	.164	1	2.891	.091	.029	1.5	.143	.806	.001
Negative symptoms	1.5	25.791	**.001**	.208	1	.293	.590	.003	1.5	2.822	.078	.028
Disorganization	1.5	30.074	**.001**	.235	1	1.451	.231	.015	1.5	1.012	.345	.010
Affect	1.5	55.936	**.001**	.363	1	1.003	.319	.010	1.5	.244	.717	.002
Resistance/Excitement-activity	1.6	7.779	**.002**	.074	1	.835	.363	.008	1.6	.349	.655	.004
PANSS total score	1.5	41.232	**.001**	.296	1	1.471	.228	.015	1.5	1.224	.289	.012
SOFAS score	1.6	113.355	**.001**	.552	1	.403	.527	.004	1.6	.644	.496	.007
Equivalent AP dose (mg/day)	1.8	3.873	**.026**	.038	1	.821	.367	.008	1.8	.414	.642	.004
Variable (group effect)	EEM (SE)											
	CHR-P +			CHR-P-								
T0 HoNOS Behavioral problems	2.456 (.260)			2.548 (.302)								
T1 HoNOS Behavioral problems	.982 (.202)			1.381 (.235)								
T2 HoNOS Behavioral problems	.860 (.192)			1.071 (.224)								
T0 HoNOS Impairment	1.741 (.191)			1.619 (.224)								
T1 HoNOS Impairment	1.190 (.174)			1.048 (.205)								
T2 HoNOS Impairment	1.224 (.153)			.643 (.180)								
T0 HoNOS Psychiatric symptoms	9.000 (.418)			8.095 (.491)								
T1 HoNOS Psychiatric symptoms	5.741 (.457)			5.000 (.537)								
T2 HoNOS Psychiatric symptoms	4.845 (.464)			4.119 (.545)								
T0 HoNOS Social problems	6.069 (.404)			4.595 (.474)								
T1 HoNOS Social problems	4.517 (.389)			3.048 (.457)								
T2 HoNOS Social problems	3.966 (.352)			2.286 (.414)								
T0 HoNOS total score	19.351 (.813)			16.857 (.947)								
T1 HoNOS total score T2 HoNOS total score	12.386 (.959) 10.912 (.932)			10.476 (1.117) 8.119 (1.088)								
T0 PANSS Positive symptoms	11.138 (.552)			9.929 (.649)								
T1 PANSS Positive symptoms	8.983 (.502)			8.095 (.590)								
T2 PANSS Positive symptoms	8.931 (.512)			7.643 (.602)								
T0 PANSS Negative symptoms	18.500 (1.101)			19.095 (1.294)								
T1 PANSS Negative symptoms	16.362 (1.099)			15.619 (1.292)								
T2 PANSS Negative symptoms	15.672 (.989)			13.405 (1.163)								
T0 PANSS Disorganization	16.345 (.837)			15.714 (.984)								
T1 PANSS Disorganization	14.224 (.788)			12.905 (.926)								
T2 PANSS Disorganization	13.586 (.684)			11.667 (.804)								
T0 PANSS Affect	15.138 (.711)			14.619 (.835)								
T1 PANSS Affect	11.552 (.586)			10.786 (.689)								
T2 PANSS Affect	10.828 (.589)			9.667 (.692)								
T0 PANSS Resistance/Excitement-activity	7.569 (.436)			7.238								
T1 PANSS Resistance/Excitement-activity	6.638 (.413)			6.238								
T2 PANSS Resistance/Excitement-activity	6.724 (.426)			5.929								
T0 PANSS total score	70.862 (2.677)			68.905 (3.146)								
T1 PANSS total score	59.793 (2.840)			55.690 (3.338)								
T2 PANSS total score	57.759 (2.878)			50.119 (3.382)								
T0 SOFAS score	48.585 (1.103)			48.488 (1.255)								
T1 SOFAS score	59.585 (1.850)			61.683 (2.104)								
T2 SOFAS score	63.264 (1.798)			65.268 (2.044)								
T0 equivalent AP dose (mg/day)	3.534 (.311)			3.800 (.365)								
T1 equivalent AP dose (mg/day)	2.379 (.328)			2.943 (.385)								
T2 equivalent AP dose (mg/day)	2.052 (.230)			2.252 (.270)								

As all Mauchly’s tests of sphericity are statistically significant (p < 0.05), Greenhouse–Geisser corrected degrees of freedom to assess the significance of the corresponding F value are used. ANOVA = analysis of variance; CHR-P = Clinical High Risk for Psychosis; CHR-P + = CHR-P individuals with previous specialist contact; CHR-P- = CHR-P individuals without previous specialist contact; df = degrees of freedom; F = F statistic value; p = statistical significance; η^2^ = partial eta squared; HoNOS = Health of the Nation Outcome Scale; PANSS = Positive And Negative Syndrome Scale; SOFAS = Social and Occupational Functioning Assessment Scale; EMM = Estimated Marginal Mean; SE = Standard Error; T0 = baseline assessment time; T1 = 1-year assessment time; T2 = 2-year assessment time; AP = Antipsychotic. Equivalent AP dose = equivalent dose of risperidone (mg/day). Statistically significant p values are in bold

## Discussion

Exploring psychiatric antecedents in youths at CHR-P remains a crucial opportunity to better describe and define their clinical trajectories, especially in prognostic terms (Polari et al., 2018). Very few investigations systematically examining this topic and there’s an overall lack of knowledge about child/adolescent prodromal syndromes in CHR-P teens, even though it has been frequently reported that several clinical risk factors for psychosis occur during childhood and adolescence (Raballo et al., 2022). In fact, this knowledge gap prevents the development of truly effective EIP interventions for these young clinical populations, resulting in treatment delay and worsening prognosis (Pelizza et al., 2024b). Furthermore, neglecting any psychiatric history in early psychosis patients may also lead to “one-off” (often non-specialist) care, to an increase in the duration of untreated psychosis or DUPrs, and to the fragmentation/discontinuity in specialist treatments (Catalano et al., 2025).

In this examination, approximately 56% of CHR-P participants had a past contact with mental healthcare services before entering the PARMS protocol. Specifically, most of them (85.3%) showed psychiatric antecedents but without current retention in care within specialized centers (care discontinuity), i.e., presenting to the PARMS program as new specialist access. Only 15% of CHR-P participants were directly referred to our EIP service by other mental healthcare centers within a generalist-to-specialized care program transition (care continuity). These findings highlight a critical issue for clinical practice: a relevant portion of youths who will later develop CHR-P mental states lacks sufficient monitoring for effective psychosis prevention and early detection within help-seeking behaviors in a crucial age range (Signorini et al., 2017). In this sense, it is important to underline the need to train psychiatrists and psychologists working in general and “low-threshold” mental health services on EIP (as well as general practitioners and emergency physicians), in order to avoid underestimating patients’ requests for help and to build a solid knowledge of a specific and shared prodromal psychopathology that can precede the onset of psychosis and other severe mental illnesses (Gammino et al., 2025). Furthermore, it is important to emphasize the need to implement early care pathways aimed at reducing the duration of help-seeking behavior and promoting appropriate access to specialist EIP services, particularly through primary care, emergency departments, and generalist mental health centers (Skrobinska et al., 2024). In this respect, the recent paradigm shift from EIP to “Youth Mental Health” (YMH) services might help to overcome some potential diagnostic delay factors (such as misdiagnosis, diagnostic underestimation of APS, considering sub-threshold psychotic features as secondary to other mental problems, including substance abuse) and to promote timely CHR-P detection, to reduce the DUPrS, and to improve long-term outcomes (Uhlhaas et al., 2023).

Additionally, in a non-negligible portion (41.8%) of CHR-P + participants, care discontinuity interested the transition age and the care transition from CAMHS to AMHS. This is not surprising considering the low rate of service engagement as care continuity within CAMHS-to-AMHS transition reported in young populations with severe mental illness (Pelizza et al., 2025d). In this respect, previous investigation examining care continuity in clinical samples with mental disorder reported that young people referred for treatment from CAMHS to AMHS are less likely to receive a discharge diagnosis of hyperkinetic and pervasive developmental disorders (Maurice et al., 2022). Based on this evidence, some authors proposed the shift towards a neurodevelopmental perspective on the development of psychosis (Gur et al., 2025) and the potential role of neurodevelopmental disorders (especially autism spectrum disorders and ADHD) as predictors of psychosis conversion (Gering et al., 2021; Guerrera et al., 2024).

Furthermore, the European MILESTONE survey (Gerritsen et al., 2022) reported that the current organization of mental health services along the pediatric/adult care model irremediably fails to match the natural course of emerging severe mental illnesses in youths, especially psychosis. Indeed, individuals aged 12–25 years have the highest incidence of severe psychiatric disorders across the lifespan, while also having the worst service engagement with CAMHS and AMHS compared to other age groups, with obvious consequences in terms of care discontinuity, unmet needs, and under-treatment (Poletti et al., 2022b). Therefore, we can no longer postpone a radical review of the architecture and resourcing of health care for youths in transition from childhood to adulthood (Raballo et al., 2017). In particular, it is absolutely necessary to strengthen those health pathways that may facilitate the transition and continuity of care between adolescent and adult services (such as YMH centers, functional teams that provide joint support from both CAMHS and AMHS operators) (Hartmann et al., 2019; McGorry et al., 2024).

Compared to the few published data on this topic, our baseline prevalence rate of past specialist contact is positioned in the highest values observed in other comparable studies. In this regard, some authors reported rates of psychiatric antecedents ranging from 25 to 37% in samples at high genetic risk for psychosis (Hans et al., 2000), while others described a prevalence of about 50% for mood and neurodevelopmental disorders during prodromal phase of psychosis (Meyer et al., 2005; Rosen et al., 2006; Mazzoni et al., 2009). These epidemiological discrepancies could be due to differences in the mean age at presentation and severity levels of clinical features. In this respect, our CHR-P sample was older compared to those recruited in previous research and did not exclusively include minors. However, overall considered, our findings confirm the importance of carefully monitoring help-seeking behavior in youths manifested in their early 20, especially in terms of psychosis prevention (Scott et al., 2024).

In this examination, the main psychiatric antecedents before entering the PARMS program in the CHR-P + subsample were anxious-depressive disorders (37.8%), learning disorder (27.4%), and conduct disorder (22.1%, including hyperkinetic disorders), with the last two diagnoses formulated within a previous contact with CAMHS. Our high rates of past anxiety and conduct disorders are in line with what was reported in the case series described by Mazzoni and co-workers (2009), who identified separation anxiety and oppositional/defiant disorder as main childhood-onset prodromes to psychosis, together with a high proportion of neurodevelopmental disorders (especially ADHD). In this respect, only two of our CHR-P participants had ADHD as specific previous diagnosis, and one had an autism spectrum disorder. Therefore, we could not examine differences between these specific psychiatric antecedents because this would likely have compromised the statistical power of our analyses. Future studies exploring these specific diagnostic entities in larger CHR-P populations are recommended.

In this investigation, we also notably found a high prevalence of previous learning disturbances. Therefore, also these diagnoses could be considered as specific antecedents of psychotic risk to be monitored in the developmental age. In this regard, some researchers formerly conceptualized psychosis also as a learning disorder, specifically as a disease generated by alterations in cognition systems (Ivleva et al., 2012; Tamminga, 2013). Indeed, it was reported that the hippocampus is altered in patients with early psychosis, with associated impairment in associative memory and learning processes (McHugo et al., 2019). Therefore, professionals working with children manifesting learning impairment should be made aware of this potential clinical long-term connection and monitor these individuals over time, also in terms of psychosis prevention.As for clinical characteristics at presentation, our CHR-P + participants showed longer DUPrS compared to CHR-P- peers. This further suggests the crucial connection of psychological suffering between past and current mental disorder and how care discontinuity may represent a critical delay in early intervention in psychosis, especially in the transition from adolescence to adulthood. Moreover, DUPrS extension represents one of the most negative prognostic factors for EIP treatment outcomes, critically prolonging the patient’s suffering and reducing the chance of effectiveness of later specialized EIP interventions. In other words, longer DUPrS has been demonstrated to be a crucial predictor related to poorer prognosis in early psychosis (Murden et al., 2024) and implementing EIP services capable of decreasing it represents one of the most important objectives to promote favorable long-term outcomes (Salazar de Pablo et al., 2024).

In this investigation, having psychiatric antecedents was associated to a higher 2-year incidence rate of psychosis transition. This finding supports the role of previous mental disorder as adverse prognostic factor, potentially favoring conversion to psychosis. This negative prognostic meaning is also confirmed by our longitudinal evidence related to higher social problems (as measure with the HoNOS) and HoNOS total scores in the CHR-P + subgroup compared to CHR-P- subjects across the whole follow-up period. Specifically, the HoNOS results further suggest that CHR-P with past contact with mental healthcare services are more likely to presents poorer long-term outcome levels and to manifest prolonged and more severe social functioning over time, maybe as consequence of a putative less effective response to our specialized EIP treatments.

However, the presence of psychiatric antecedents was also associated with a lower 2-year incidence rate of service disengagement. In this perspective, the presence of psychiatric antecedents could be considered as an index of greater psychopathological severity and more severe clinical trajectories by mental health professional working within our EIP service. This may have led to stronger retention in care and more careful monitoring of these CHR-P + subgroup.

### Limitations

This investigation also has noteworthy limitations. First, information on previous specialist contact and psychiatric antecedents was collected retrospectively and registered from medical records. This method is prone to information bias, as the data in the medical records may be inaccurate or incomplete.

Second, there was no knowledge about potential previous primary care treatments. In this regard, some patients could have received pharmacological treatment for potential, sub-threshold anxious-depressive symptoms from their general practitioners. This may also have resulted in an underestimation of CHR-P participants with mental health antecedents.

Third, our examination was limited to 2 years of follow-up. Our findings are thus comparable only with research having longitudinally similar designs. Furthermore, this could lead to exclusion of a subpopulation with clinical characteristics that further delay the diagnosis, which may show lower help-seeking behavior and may suffer greater damage from prolonged DUPrS. Therefore, future studies with longer follow-up duration are needed.

Forth, we did not dig into DSM-5 diagnostic categories outside of CHR-P + vs. CHR-P- subgroups. Although it may be relevant to examine differences across specific psychiatric antecedents (especially neurodevelopmental disorders), this would likely have compromised the statistical power of our analyses, given the large number of diagnostic categories detected, their low prevalence, and the relatively small sample size. Therefore, future studies exploring specific diagnostic entities (such as ADHD and autism spectrum disorders) in larger CHR-P populations are recommended. Moreover, we did not explore the broad-band internalizing/externalizing dichotomy and how this classification may differentially affect outcomes of interest in CHR-P individuals (e.g., psychosis transition, service engagement). Because previous evidence has shown that different disorders confer varying degrees of risk for psychosis phenotypes, we’ll investigate this interesting dichotomy in future analyses.

Fifth, overall considered, our results were partially influenced by the choice of our nosographic classification and the credibility of reported psychiatric antecedents, including the potential under-reporting or lack of information on the prevalence of neurodevelopmental disorders within our CHR-P sample. In particular, another crucial question remains open: i.e., whether these antecedents represent prodromal symptoms or are independent diagnostic entities. However, in this investigation, we were interested in the psychiatric antecedents as previous diagnoses/categories as they are described in the common classification system (i.e., the DSM-5). To ensure greater statistical power to our analyses, we used a unitary classification to group similar diagnoses in fairly homogeneous diagnostic subgroups. We were aware that this could be an approximate and incomplete classification, but it seemed to be sufficient to us for an initial, exploratory analysis.

Finally, this investigation was conducted entirely in Italy as single-center study. Our results could represent outcomes related to the organization of Italian public mental health services. An international multicenter research is thus needed to clarify whether and how different health care organizations may lead to different trajectories of pre-psychotic symptoms and syndromes.

## Conclusions

The findings of this study showed that most (55.9%) of CHR-P participants showed psychiatric antecedents and a past contact with mental healthcare services. Specifically, 53.7% of these diagnoses (i.e., learning disorder, conduct disorder, and anorexia nervosa) were formulated within CARMS. Moreover, in 85.2% of these cases, we documented a care discontinuity. Greater effort should thus be made to identify psychiatric antecedents of psychosis in both YMH services and primary care, as well as to favor and implement care continuity during child-adult transition, and to better manage the interfaces among specialist mental healthcare services, also in order to reduce the DUPrS. Indeed, CHR-P individuals with past contact showed to have higher risk of psychosis transition and poorer functioning and outcome scores over time. In this sense, previous specialist contact may be considered as a predictor of unfavorable prognosis and poor response to treatment.

Lorenzo Pelizza and Fabio Catalano: conceptualization and study design; Lorenzo Pelizza, Fabio Catalano, and Simona Pupo: literature search; Lorenzo Pelizza, Emanuela Leuci, and Emanuela Quattrone: data collection and curation; Lorenzo Pelizza and Fabio Catalano: Formal analysis; Lorenzo Pelizza and Fabio Catalano: writing – original draft; all authors: writing – review & editing.

## Supplementary Information

Below is the link to the electronic supplementary material.

ESM 1 (DOCX 49.6 KB)

## Data Availability

The data that support the findings of this research are available on reasonable request from the corresponding author. The data are not publicly available due to privacy and/or ethical restrictions.
