# Supplementary material for: Psychiatric Antecedents in Adolescents at Clinical High Risk for Psychosis: Insights from the “Parma At-Risk Mental States” Follow-up Program
Source: Res Child Adolesc Psychopathol. 2025 Aug 30;53(11):1713–30. doi: 10.1007/s10802-025-01368-0 (PMC12586412; doi:10.1007/s10802-025-01368-0)
Supplement: Supplementary file 1 — (DOCX 49.6 KB) [file 10802_2025_1368_MOESM1_ESM.docx]

Figure S1. Mixed-design ANOVA results: between-group comparisons on profile plots of statistically relevant HoNOS scores across the 2-year follow-up period in the two CHR-P subgroups.


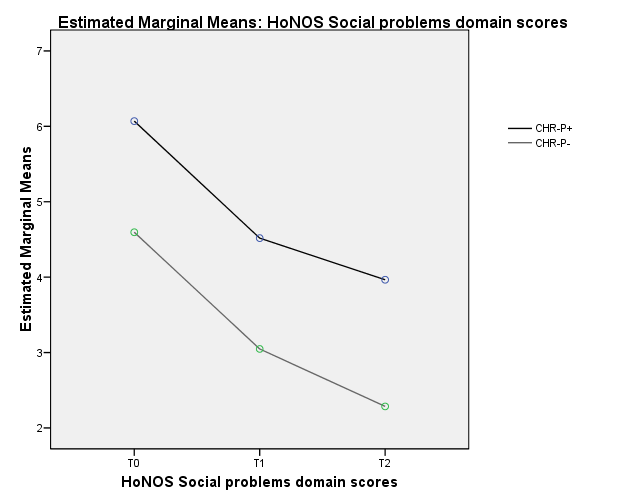


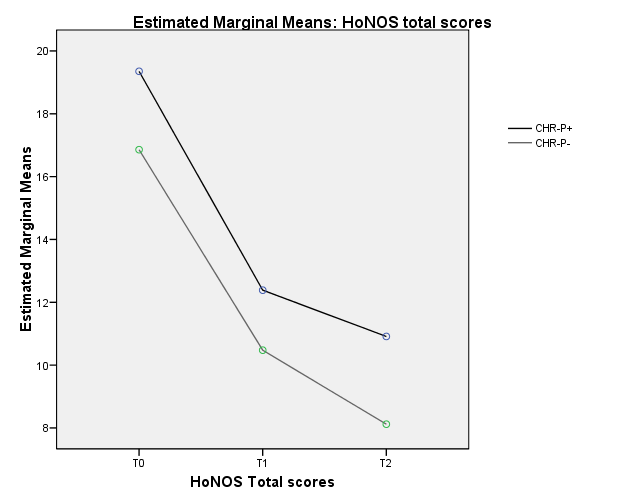


Note. CHR-P = Clinical High Risk for Psychosis; CHR-P+ = CHR-P individuals with previous specialist contact; CHR-P- = CHR-P individuals without previous specialist contact; HoNOS = Health of the Nation Outcome Scale; T0 = baseline assessment; T1 = 1-year assessment time; T2 = 2-year assessment time.
